# Exploiting Natural Killer Cell Engagers to Control Pediatric B-cell Precursor Acute Lymphoblastic Leukemia

**DOI:** 10.1158/2326-6066.CIR-21-0843

**Published:** 2022-03-03

**Authors:** Natalia Colomar-Carando, Laurent Gauthier, Pietro Merli, Fabrizio Loiacono, Paolo Canevali, Michela Falco, Federica Galaverna, Benjamin Rossi, Frédéric Bosco, Mélody Caratini, Maria Cristina Mingari, Franco Locatelli, Eric Vivier, Raffaella Meazza, Daniela Pende

**Affiliations:** 1 Laboratory of Immunology, IRCCS Ospedale Policlinico San Martino, Genova, Italy.; 2 Department of Experimental Medicine, University of Genoa, Genova, Italy.; 3 Innate Pharma, Marseille, France.; 4 Department of Hematology/Oncology and Cell and Gene Therapy, IRCCS Ospedale Pediatrico Bambino Gesù, Roma, Italy.; 5 Laboratory of Clinical and Experimental Immunology, Integrated Department of Services and Laboratories, IRCCS Istituto Giannina Gaslini, Genova, Italy.; 6 Department of Gynecology/Obstetrics and Pediatrics, Sapienza University, Roma, Italy.; 7 Aix Marseille University, CNRS, INSERM, CIML, Marseille, France.; 8 APHM, Hôpital de la Timone, Marseille-Immunopôle, Marseille, France.

## Abstract

Natural killer (NK) cells represent a promising cell type in antitumor immunotherapy for efficacy and safety, particularly in the treatment of hematologic malignancies. NK cells have been shown to exert antileukemia activity in the context of haploidentical hematopoietic stem cell transplantation (haplo-HSCT). Products have been developed to boost the activation of NK cells only when cross-linked by tumor cells, avoiding any off-target effect. Here, we tested the *in vitro* effect of different NK-cell engagers (NKCE), which trigger either NKp46 or NKp30 together with CD16A, and target either CD19 or CD20 to induce killing of pediatric B-cell precursor acute lymphoblastic leukemia (BCP-ALL). Target cells were NALM-16 and MHH-CALL-4 cell lines and four primary leukemias, while effector cells were resting NK cells derived from healthy donors and pediatric patients with leukemia after αβT/B-depleted haplo-HSCT. The NK cell–resistant MHH-CALL-4 was efficiently killed using all NKCEs. Boosting of NK activity against MHH-CALL-4 was also evident by degranulation and IFNγ production. Because of the lack of CD20 and high expression of CD19 on primary BCP-ALL, we focused on NKCEs targeting CD19. NKp46- and NKp30-based NKCEs displayed similar potency at inducing NK-cell activity, even when challenged with primary BCP-ALL blasts. Their efficacy was shown also using NK cells derived from transplanted patients. NKCE-induced activation against BCP-ALL can override HLA-specific inhibitory interactions, although the strongest response was observed by the alloreactive NK-cell subset. These data support the therapeutic use of NKp46/CD16A/CD19-NKCE to fight refractory/relapsed leukemia in pretransplantation or posttransplantation settings.

## Abstract (precis)

NK-cell engagers (NKCE) can enhance NK cell–mediated killing of pediatric BCP-ALL, particularly when using a CD19-targeting NKCE that engages NKp46 together with CD16A. The data support the clinical use of NKCEs to treat patients with BCP-ALL.

## Introduction

Natural killer (NK) cells are essential components of innate immunity and are involved in the first line of antitumor defense, preventing tumor growth and spreading (1, 2). Two subsets of NK cells can be identified: (i) CD56^dim^CD16^+^ NK cells, the major subset in peripheral blood (PB), are well equipped with lytic granules containing perforin and granzyme B and display strong cytotoxic activity; and (ii) CD56^bright^CD16^low/−^ NK cells are poorly cytotoxic and produce IFNγ and TNFα upon cytokines stimulation. CD16A (FcγRIIIa), the NK-cell receptor of IgG, is a potent activating receptor mediating antibody-dependent cellular cytotoxicity.

NK-cell effector function is finely regulated by the balance between activating or inhibitory signals transmitted by an array of receptors upon engagement with specific ligands (3). “On” signals induce the cross-talk with other cell types, and they prevail when NK cells encounter pathologic cells overexpressing stress-inducible ligands. This event is exemplified by NKG2D triggering receptor upon recognition of MICA/B and UL16 binding proteins (ULBP). Specifically expressed on NK cells are natural cytotoxicity receptors (NCR; i.e., NKp46, NKp30, and NKp44), which represent major activating receptors involved in tumor cell lysis (4). NKp46 (5) and NKp30 (6) are present on most, if not all, resting and cultured NK cells, whereas NKp44 (7) is acquired after activation. Although several molecules have been described to interact with NCRs (8), only a few membrane-bound ligands have been identified: B7-H6 (9), and HLA-DP401 (10). Another activating receptor shown to be involved in NK-cell/tumor interaction is DNAM-1, which recognizes CD155/PVR (poliovirus receptor) and CD112/Nectin-2 (11). For “off” signals, inhibitory receptors include killer Ig-like receptors (i.e., iKIR), which recognize epitopes shared by distinct groups of HLA-A, -B, or -C allotypes, also identified as KIR ligands (KIR-L). Briefly, KIR2DL1 is specific for HLA-C allotypes that share lysine 80 (C2 epitope); KIR2DL2/L3 recognize with high-affinity HLA-C allotypes sharing asparagine 80 (C1 epitope), HLA-B*46:01, and HLA-B*73:01, while with low affinity the C2 epitope. KIR3DL1 binds HLA-B and HLA-A molecules sharing the Bw4 public epitope (12). The C-type lectin-like CD94/NKG2A heterodimeric receptor recognizes the non-classical HLA-E (13). Activating counterparts are represented by activating KIRs (i.e., aKIR) and CD94/NKG2C.

Heterogeneous circulating NK-cell populations can be observed among different individuals. NK-cell repertoires are primarily determined by genetic factors (namely *KIR* gene repertoire and *HLA* class I genotype), but also influenced by environmental stimuli, in particular cytomegalovirus infection (14, 15). The high polymorphism of the coinherited *KIR* and *HLA* class I alleles creates diverse compound genotypes, that are translated into phenotypes following the rules of NK-cell education (16). During NK-cell development, each NK cell becomes fully competent only if it expresses at least one inhibitory receptor recognizing self-HLA class I. This process ensures the capacity to discriminate between healthy autologous cells (“self-tolerance”) and pathologic cells that have lost HLA class I molecules (“missing-self recognition”), which can occur in cancer cells (17).

In allogeneic hematopoietic stem cell transplantation (HSCT) settings, through missing self-recognition, NK cells can be alloreactive, according to the expression of iKIR(s) specific for KIR-L(s) present in the donor and absent in the allogeneic cells. Taking advantage of NK-cell alloreactivity, primarily the experience of HSCT from an HLA-haploidentical donor (haplo-HSCT) to treat patients with high-risk leukemia, has led to the notion that NK cells, the first lymphocyte subset reconstituting after HSCT, display antileukemia activity (graft-versus-leukemia, GvL) and, different from alloreactive T cells, do not cause GvHD (18–20). These clinical results have encouraged the consideration of NK cells as an attractive product in terms of efficacy and safety for adoptive cellular immunotherapy. Several strategies have also been developed to improve NK-cell effectiveness and enhance their antitumor activity, particularly for treating hematologic malignancies (21–23).

To specifically redirect NK-cell killing to neoplastic cells, molecules termed NK-cell engagers (NKCE) have been produced with the aim of improving immunologic synapse formation and cell activation (23, 24). Bispecific killer cell engagers (BiKE; ref. 25), composed of two single-chain variable fragments, engage CD16A and target tumor-associated antigens (TAA). Trispecific killer engagers (TriKE), incorporating a modified human IL15 cross-linker, have been produced and shown to be active in preclinical models (26, 27). The 161533 TriKE is currently being tested in a phase I/II clinical trial (NCT03214666) for the treatment of CD33^+^ myeloid malignancies. A second-generation CD33-targeting TriKE has been also produced and shows improved functionality *in vitro* and in preclinical mouse models and is potentially more efficacious in clinics (28). A tetravalent bispecific antibody that binds CD30 on tumor cells and CD16A on NK cells (AFM13) has also been tested in a phase I trial for patients with relapsed/refractory Hodgkin lymphoma (29).

In addition to CD16A, the engagement of other triggering NK-cell receptors, such as NKG2D and NCRs, can be extremely valuable (24, 30, 31). In line with the notion that full activation of resting NK cells requires the coengagement of distinct activating receptors (32, 33), trifunctional NKCEs referred to as antibody-based NK-cell engager therapeutics (ANKET), which coengage NKp46 and CD16A on NK cells and bind an antigen on tumor cells (NKp46/CD16A/TA), have been produced. These NKCEs show more potent activity than therapeutic mAbs (e.g., anti-CD20 rituximab and obinutuzumab) and effectiveness in the control of tumor growth in mouse models (34). We report here the *in vitro* analysis of NKCEs that trigger either NKp46 or NKp30, in addition to CD16A, and target CD19 or CD20 to induce killing of pediatric B-cell precursor acute lymphoblastic leukemia (BCP-ALL), which represents the most common childhood malignancy. Despite the improvement in the treatments, approximately 15% of children with BCP-ALL relapse after frontline chemotherapy (35). Currently, different strategies to prevent further recurrence in patients with BCP-ALL represent an important clinical challenge. NKp46/CD16A/CD19 NKCE proved effective in enhancing NK-cell activity, even towards primary BCP-ALL blasts. Their efficacy was also shown using NK cells derived from pediatric patients with leukemia after αβT/B-depleted haplo-HSCT. These data pave the way for the development of ANKETs in posttransplantation settings in patients with BCP-ALL.

## Materials and Methods

### Healthy donors, patients with leukemia, and cell separation

All samples were obtained following written informed consent from donors and patient parents/legal guardians in accordance with the Declaration of Helsinki. Buffy coats from healthy donors (*n* = 14) were provided by the blood transfusion center of IRCCS Ospedale Policlinico San Martino (Genoa, Italy), following approved internal operational procedures (IOH78). PB samples were also obtained from donors (*n* = 11) and pediatric patients with leukemia (*n* = 22) at different timepoints (1, 2, 3, 12 months) after αβT/B-depleted haplo-HSCT at IRCCS Ospedale Pediatrico Bambino Gesù (OPBG, Rome, Italy). This clinical trial was approved by the Institutional Review Board (Ethical Committee) of OPBG (TCR αβ haplo-HSCT-OPBG; Protocol no. 424/2011) and registered at ClinicalTrial.gov (NCT01810120). All donors for haplo-HSCT were typed for *HLA* class I and analyzed for KIR-L and *KIR* genotype, as described previously (36). Primary BCP-ALL blasts were derived from PB or bone marrow (BM) of pediatric patients at diagnosis (*n* = 4) in IRCCS Ospedale Pediatrico Bambino Gesù. PB or BM mononuclear cells (PBMC, BMMCs, respectively) were isolated by Lympholyte-H (Cedarlane Laboratories) density-gradient centrifugation, and phenotypically characterized by immunofluorescence as described below. NK-cell isolation was performed using RosetteSep human NK-cell enrichment cocktail (catalog no.: 15065; StemCell Technologies) following the manufacturer's instructions, obtaining a purity of ≥90%. All samples used in this study were collected from 2019 to 2021, and the cells were cryopreserved in FBS (Euroclone) containing 10% DMSO (Panreac Quimica).

### Cell lines and KIR-L analysis

The cell lines were cultured in RPMI1640 (Lonza) supplemented with 10% FBS (with the exception of 20% FBS for MHH-CALL-4; Euroclone), 2 mmol/L l-glutamine (Lonza), and 100 U/mL penicillin-streptomycin (Lonza) in 5% CO_2_ incubator at 37°C. NALM-16 (catalog no.: ACC-680) and MHH-CALL-4 (catalog no.: ACC-337), two pediatric BCP-ALL cell lines, were obtained from DSMZ in 2014 and 2020, respectively. The erythroleukemia K562 and the lung carcinoma A549 cell lines were certified in 2020 by short tandem repeat analysis performed by Italian cell line collection (ICLC, www.iclc.it) in accordance to profiles published on Expasy - Cellosaurus (https://web.expasy.org/cellosaurus/) and Clima 2 (http://bioinformatics.hsanmartino.it/clima2/index.php). All cell lines were routinely screened for *Mycoplasma* infection by PCR analysis, and used within four passages in culture after thawing.

DNA was extracted from 5 × 10^6^ MHH-CALL-4 cells using the QIAamp DNA Blood Mini kit (Qiagen), and DNA concentration was adjusted to 20 ng/μL. KIR-Ls were analyzed using Olerup KIR HLA ligand kit (GenoVision), based on a sequence-specific primer (SSP)-PCR approach, and following the manufacturer's instructions. PCR reactions were performed using the Bio-Rad T100 Thermal Cycler.

### Production and purification of NKCEs targeting CD19 or CD20

All the purified molecules were stored in 1× PBS and analyzed to check for the absence of aggregates by ultra-performance liquid chromatography (ACQUITY UPLC H-Class Bio; ACQUITY UPLC PDA Detector; Waters) using the ACQUITY UPLC Protein BEH SEC column (200 Å, 1.7 μm, 4.6 mm × 150 mm; Waters) and of endotoxins by kinetic chromogenic assay (KCA Endochrome-K; Charles River). NKp46- and NKp30-NKCEs targeting CD19 and CD20 were generated under the previously described multifunctional format NKCE-2 (34). NKCE-2 format is constituted by the assembly of three polypeptide chains with the following domain arrangements, respectively: (ABD_L)_1_-C_K_-H-CH2-CH3, (ABD_H)_1_-CH1-H-CH2-CH3-(ABD_H)_2_-C_K_, and (ABD_L)_1_-CH1. (ABD_H)_1_ and (ABD_L)_1_ are the antibody heavy and light chain variable domains that bind to the target antigen on cancer cells. (ABD_H)_2_ and (ABD_L)_2_ are the antibody heavy and light chain variable domains that bind to NKp46 or NKp30 on NK cells. CH1, CH2, CH3, and H are the human IgG1 constant domains and hinge. C_K_ is the human kappa light chain constant domain. NKCEs targeting CD19 were built using heavy and light chain variable domains of the CD19 antibody MT-103 (37). NKCEs targeting CD20 were built using heavy and light chain variable domains of the CD20 antibody GA101 (38). NKCEs engaging NKp46 or NKp30 were built using heavy and light chain variable domains of the NKp46 antibody NKp46-1 (34) and NKp30 antibody Az20 (6), respectively. Isotype control NKCEs were built using heavy and light chain variable domains derived from the Cn2 antibody 9004G (39).

The sequences encoding for the three different fragments of each multispecific molecule (40) were synthesized by Eurofins genomics and inserted into the pTT-5 vector from National Research Council Canada. The insertions were performed between the HindIII and BamHI restriction sites. Expression vectors were used to cotransfect EXPI-293F cells (Life Technologies, Thermo Fisher Scientific) at 37°C, 5% CO_2_, 150 rpm, in the presence of PolyEthyleneImine Max (PEI; Polysciences Inc) with a DNA/PEI ratio of 1/1 (w/w). Cells were used to seed culture flasks at a density of 1 × 10^6^ cells/mL and were cultured in EXPI293 medium (Gibco, Thermo Fisher Scientific) supplemented with valproic acid (final concentration 0.5 mmol/L; Sigma-Aldrich), D(+) glucose monohydrate (4 g/L; Honeywell Fluka), and tryptone N1 (0.5%; Organo Technie) 1 day after transfection. The supernatants were harvested after 6 days and passed through a 0.22-μm Stericup (Merck Millipore) filter. Multispecific molecules were purified with rProtein A Sepharose Fast Flow (GE Healthcare; 250 μL/50 mL supernatant), eluted with 0.1 mol/L sodium citrate buffer at pH 3, and immediately neutralized with 1 mol/L Tris pH 8. The proteins were then dialyzed overnight against 1× DPBS (not supplemented with calcium nor magnesium; Gibco, Thermo Fisher Scientific) with Slide-A-Lyzer dialysis cassettes (Thermo Fisher Scientific) at 4°C. Samples were then concentrated with Amicon Ultra 15 10K (Merck Millipore) to 10 mg/mL before loading on an S200 Increase 10/300GL column (GE Healthcare). The proteins yielding a peak at the expected size (150 kDa) were harvested.

Alternatively, NKCE molecules were also purified by ion-exchange chromatography on a MonoS 4.6/100PE column (GE Healthcare) using an Aktä Pure 25 M (GE Healthcare). Before chromatography, each sample was dialyzed overnight against 25 mmol/L phosphate buffer, pH 6.2 (KH_2_PO_4_/Na_2_HPO_4_; Sigma-Aldrich). Sample loading on the column was then performed at 2 mL/minute, with an elution linear gradient of salt from 0–200 mmol/L NaCl (Sigma-Aldrich). The peak of interest was determined according to the analysis performed on SDS PAGE NuPAGE Novex BisTris 4%–12% gel (Invitrogen, Thermo Fisher Scientific) under reduced and non-reduced conditions with Instant Blue (Euromedex) Coomassie staining. The proteins were then dialyzed overnight against 1× DPBS (not supplemented with calcium nor magnesium; Gibco, Thermo Fisher Scientific) with Slide-A-Lyzer dialysis cassettes (Thermo Fisher Scientific) at 4°C. Finally, samples were then concentrated with Amicon Ultra 15 10K (Merck Millipore) at least to 1 mg/mL.

### Cytofluorimetric analysis

Leukemia cell lines were analyzed by indirect immunofluorescence using CD19 and CD20 mAbs, followed by PE-conjugated anti-IgG1 secondary reagent (1:5,000; catalog no.: 1070-09; Southern Biotech). The threshold for high/low expression of CD19 and CD20 was considered 90% positive cells. Primary BCP-ALL samples were analyzed using CD19-PE-Cy7, CD20-V450, CD3-BV510, CD45-APC Vio770 mAbs. HLA class I expression on leukemia cell lines and primary blasts was evaluated by indirect immunofluorescence using the W6/32 mAb and FITC-conjugated anti-IgG secondary reagent (1:2,500; catalog no.: 1030-02; Southern Biotech). For primary leukemia, this staining was followed by two washes and incubation with CD45-APC Vio770 mAb to discriminate the healthy counterpart from leukemia blasts. The staining index (SI) was defined as the difference between the median fluorescence intensity of cells stained with the relevant mAb and that of the negative control divided by two times the SD of the negative control. The threshold of SI = 5 was considered for high/low HLA class I expression.

Surface phenotype of NK cells was performed on PBMCs (gating CD3^−^CD56^+^ cells) from healthy donors and patients after αβT/B-depleted haplo-HSCT by multiparametric flow cytometry. For intracellular staining, surface-labeled cells were fixed and permeabilized using Cytofix/Cytoperm Fixation/Permeabilization Kit (catalog no.: 554714; BD Biosciences), washed twice with Perm/Wash Buffer 1× (BD Biosciences) and stained with anti-perforin and anti-granzyme B or the corresponding isotype control mAbs. All antibodies used in this study are detailed in Supplementary Table S1. For all the stainings, 200,000 cells were used in each sample. All the incubations with antibodies were performed at 4°C for 30 minutes; thereafter, the cells were washed with PBS (Lonza) with 2% FBS (Euroclone). Stained samples were acquired using Gallios (Beckman Coulter) or MACSQuant-analyzer (Miltenyi Biotech) and analyzed with FlowJo, Version 10.7 (BD Biosciences).

### Cytotoxicity assays

Cryopreserved or freshly isolated NK cells from either healthy donors or transplanted patients were incubated overnight with complete medium (10% FBS) and then tested in functional assays against different target cells (leukemia cell lines, primary BCP-ALL, and A549). Effector and target cells were cocultured for 4 hours at 37°C using an E:T ratio of 10:1, unless otherwise specified, with the different NKCEs at various concentrations (from 10^0^ to 10^−4^ μg/mL). In some experiments, rituximab (Rixathon, Sandoz GmbH) and cetuximab (Erbitux, Merck) were also used at the indicated concentrations. For ^51^Chromium (^51^Cr)-release assays, 10^6^ target cells (leukemia cell lines and A549) were labeled upon incubation at 37° C for 1 hour with 100 μCi of ^51^Cr-Sodium Chromate (PerkinElmer), as described previously (19, 41).

Because of new limitations in the use of radiolabeled material and difficulties of ^51^Cr labeling of primary leukemia, we additionally setup cytotoxicity assays using 7AAD/AnnexinV (7AAD/AnnV) staining. Briefly, 0.4–1 × 10^6^ resting NK cells were labeled with Cell Trace Violet (CTV, catalog no.: C34557; Thermo Fisher Scientific) at 37°C for 15 minutes, according to manufacturer's instruction. After washing in PBS, CTV-labeled NK cells were resuspended at 1 × 10^6^/mL in complete medium. 100,000 CTV-labeled NK cells were cocultured for 4 hours with 10,000 target cells (MHH-CALL-4, K562, and primary BCP-ALL) in U bottom 96-well plates (Corning Incorporated) in the presence of NKCEs at different concentrations as indicated. Wells with target cells alone were used as control. Apoptosis of target cells (gated as CTV-negative cells) was analyzed by assessing 7AAD (catalog no.: 51-68981E; BD Biosciences) and AnnV-FITC (catalog no.: BMS306FI-300; Thermo Fisher Scientific) by flow cytometry. Percentage of specific lysis was calculated following this formula:




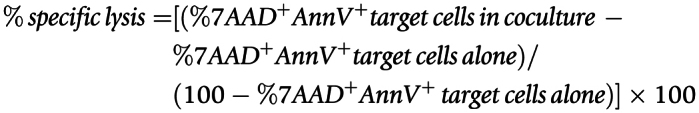




### Degranulation assay and IFN**γ** production

NK-cell activation by NKCEs against BCP-ALL cells was evaluated using CD107a expression and, in some experiments, IFNγ production by flow cytometry. Resting human NK cells from healthy donors or PBMCs from transplanted patients (primarily in the first trimester posttransplant) were cultured for 4 hours in the presence or absence of BCP-ALL cells at E:T 1:1 at 37°C in the presence of the indicated concentration of NKCEs. Golgi Stop (1:1,500; catalog no.: 554724; BD Biosciences) was added after the first hour of incubation. Thereafter, cells were washed, incubated at 4°C for 30 minutes with Live/Dead Fixable Aqua stain (1:1,000; catalog no.: 34957; Thermo Fisher Scientific), and, after washing, stained (30 minutes at 4°C) with anti-CD3-PE-CF594, -CD56-PE-Cy7, and -CD107a-FITC. Appropriate antibody combinations allowing the identification of NK-cell subsets (sKIR2DL1^+^, KIR2DL3^+^/KIR3DL1^+^, and KIR^−^NKG2A^+^) were used, and the gating strategy is shown in Supplementary Fig. S1. For the simultaneous analysis of CD107a and IFNγ expression, 6-hour cocultures were performed, and Golgi Plug (1:1,000; catalog no.: 555029; BD Biosciences) was also added after the first hour of incubation. After surface staining, as described above, cells were fixed and permeabilized with Cytofix/Cytoperm, washed twice with the Perm/Wash Buffer 1× (both from BD Biosciences), and stained with anti-IFNγ-PE. Degranulation assays were also performed using PBMCs activated in culture for 12 days with IL15 (catalog no.: 1413-C10; CellGenix) at 10 ng/mL. After a 4-hour incubation with MHH-CALL-4 cells and CD19-NKp30-NKCE (or IC-NKp30-NKCE), activated PBMCs were stained with anti-CD3-PE-CF594, anti-CD56-PE-Cy7, anti-CD107a-PE, and TCR PAN γδ-FITC. mAbs used in these assays are described in Supplementary Table S1. Samples were analyzed using a Gallios or MACSQuant-analyzer. % CD107a^+^ and IFNγ^+^ cells represent the difference between the percent of CD107a^+^ (or IFNγ^+^) NK cells cocultured with target cells and the % of CD107a^+^ (or IFNγ^+^) NK cells cultured with medium alone.

### Statistical analysis

Graphical representation and statistical analysis were performed with Prism software, Version 9.0.2 (GraphPad Software). Mann–Whitney test was used to compare two groups with non-normally distributed variables. Two-way ANOVA, followed by Tukey comparison test, was used to analyze experiments with more than two groups. Significance is indicated as: *, *P* ≤ 0.05; **, *P* ≤ 0.01; ***, *P* ≤ 0.001. *N* is the number of samples used in the experiments. The means are shown, and bars indicate SEM.

### Data availability

The data generated in this study are available upon request from the corresponding author.

## Results

### NKp46- and NKp30-NKCEs potentiate NK-cell activation against BCP-ALL

**Figure 1.**
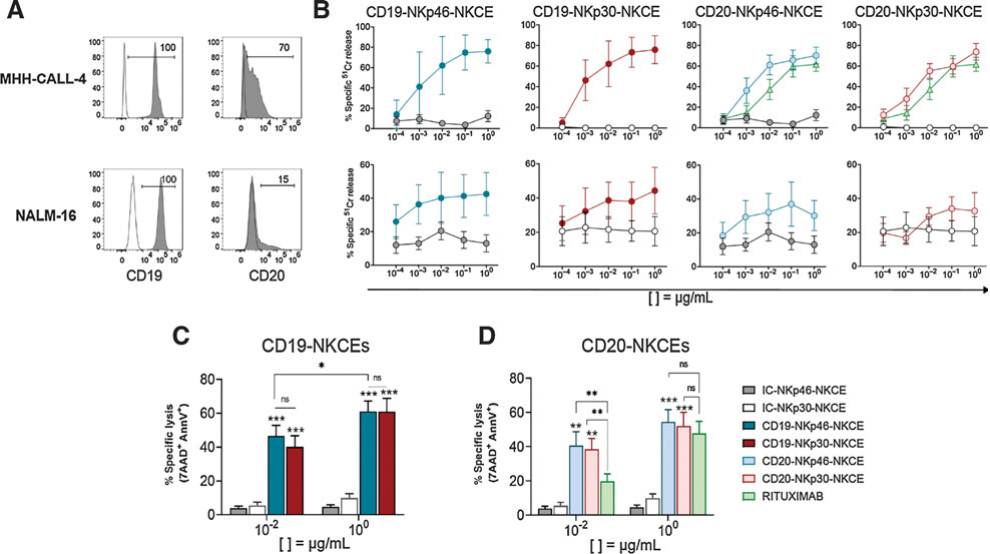
Effect of NKCEs targeting CD19 or CD20 on BCP-ALL cell lines. **A,** Two pediatric BCP-ALL cell lines were characterized for the surface expression of CD19 and CD20 via flow cytometry using specific mAbs followed by PE-conjugated anti-IgG1 secondary reagent. Numbers represent the percentage of positive cells. **B,** Comparison of cytotoxicity (^51^Cr-release assay) of resting NK cells from healthy donors against MHH-CALL-4 (top row, *n* = 2–6) or NALM-16 (bottom row, *n* = 3) cells in the presence of NKCEs at the indicated concentrations (see legend for color symbols in **D**). E:T ratios were 10:1 and 5:1 for MHH-CALL-4 cells and NALM-16 cells, respectively. Percent of specific lysis, via 7AAD/AnnV staining, of MHH-CALL-4 cells cocultured with resting NK cells from healthy donors (*n* = 4–9) in the presence of CD19-NKCEs or IC-NKCEs (**C**), and CD20-NKCEs, IC-NKCEs, or rituximab (**D**) at 10^−2^ μg/mL and 10^0^ μg/mL. Results from 6–10 independent experiments are reported. In both cytotoxicity assays, 4-hour coculture was performed. Bars show mean ± SEM. Statistical significance: *, *P* ≤ 0.05; **, *P* ≤ 0.01; ***, *P* ≤ 0.001. Two-way ANOVA followed by Tukey test was used to calculate statistical differences among the indicated NKCEs within each concentration. Mann–Whitney test was used to compare each NKCE at the two indicated concentrations.

Trifunctional NKp46 engager molecules (hereafter referred to as CD19-NKp46-NKCE and CD20-NKp46-NKCE), coengaging CD16A and targeting either CD19 or CD20, have been demonstrated to promote efficient NK cell–mediated antitumor activity in several preclinical models of lymphoma (34). Using the same trifunctional antibody format, new NKCEs engaging NKp30 instead of NKp46 and coengaging CD16A have been also produced which target CD19 (i.e., CD19-NKp30-NKCE) or CD20 (i.e., CD20-NKp30-NKCE). NKCE molecules where the anti-TAA (CD19 or CD20) was replaced with isotype control (IC-NKp46-NKCE, IC-NKp30-NKCE) were used as negative control molecules in all functional assays. We tested the *in vitro* effects of these NKCEs to potentiate NK-cell activity against leukemia cells. We selected two pediatric BCP-ALL cell lines, MHH-CALL-4 and NALM-16, which were analyzed for the expression of the relevant target antigens. Whereas high CD19 expression was detected on the cell surface of both MHH-CALL-4 and NALM-16 cells, only low CD20 was observed (Fig. 1A).

We next assessed the antileukemia efficacy of resting NK cells from healthy donors against the two BCP-ALL cell lines in the presence of different concentration of NKp46-NKCEs or NKp30-NKCEs, targeting either CD19 or CD20 (Fig. 1B). NK cell–mediated killing of both cell lines was induced using all NKCEs, starting from a concentration of 10^−3^ μg/mL and reaching the peak of activity at 10^−1^ or 10^0^ μg/mL. The effect induced by NKCEs toward MHH-CALL-4 cells was particularly evident, given the resistance of this cell line to NK-cell lysis. Conversely, NALM-16 cells displayed more susceptibility to NK-mediated killing. Consistent with the different expression of the target molecules, CD19-NKCEs were more effective than CD20-NKCEs. In parallel to CD20-NKCEs, the anti-CD20 rituximab was also tested and showed a lower effect, particularly at 10^−2^ μg/mL. In further experiments, we then focused on MHH-CALL-4 target cells and used a 7AAD/AnnV-based cytotoxicity assay and cytofluorimetric analysis, which yielded similar results as the ^51^Cr-release assays (Supplementary Fig. S2).

**Figure 2.**
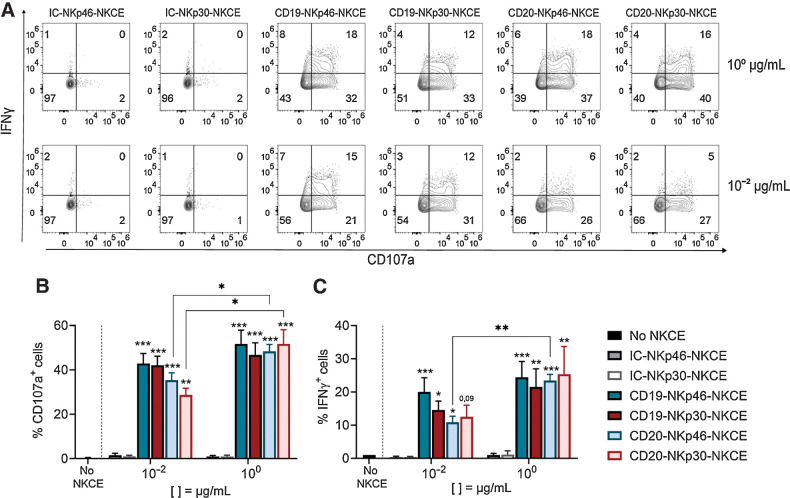
NKCEs enhance NK-cell activity against MHH-CALL-4 cell line. CD107a expression and IFNγ production by resting NK cells from healthy donors (*n* = 3–6) cocultured for 6 hours with MHH-CALL-4 cells in the absence or presence of CD19-NKCEs, CD20-NKCEs, or the control molecules (IC-NKp46-NKCE, IC-NKp30-NKCE) at 10^−2^ μg/mL and 10^0^ μg/mL. **A,** Flow cytometry of a representative experiment is shown. Numbers indicate the percentage of cells in each quadrant. CD107a expression (**B**) and IFNγ production (**C**) were performed in 3–6 independent experiments. Bars show mean ± SEM. Statistical significance: *, *P* ≤ 0.05; **, *P* ≤ 0.01; ***, *P* ≤ 0.001. Two-way ANOVA followed by Tukey test was used to calculate statistical differences among the indicated NKCEs within each concentration. Mann–Whitney test was used to compare each NKCE at the two indicated concentrations.

Thereafter, we selected two NKCE concentrations, namely 10^0^ μg/mL (optimal) or 10^−2^ μg/mL (suboptimal). We also found that NKCE engagement of either NKp46 or NKp30 was equally efficient (Fig. 1C and D). When targeting CD20, we found that CD20-NKCEs at 10^−2^ μg/mL were significantly more efficient than rituximab to induce MHH-CALL-4 killing (41 ± 6.99 for CD20-NKp46-NKCE and 39 ± 6.77 for CD20-NKp30-NKCE vs. 20 ± 13.97 for rituximab; Fig. 1D). We found that these NKCEs did not enhance the killing of cell lines lacking CD19 or CD20, namely K562 and A549 cells (Supplementary Fig. S3), demonstrating the effects were target antigen–specific. Finally, we investigated the effect of NKCEs on NK-cell activation against MHH-CALL-4 cells by analyzing CD107a degranulation and cytokine production by resting NK cells in the presence of NKCEs. Both NKp30- or NKp46-NKCEs equally displayed capacity to induce CD107a degranulation (Fig. 2A and B) and IFNγ expression (Fig. 2A and C). Targeting CD19 was more efficient than CD20 because the CD20-NKCEs showed a significantly reduced activity at the lower concentration. Altogether, these data show that both CD19-NKp46-NKCE and CD19-NKp30-NKCE potentiate NK-cell activity against BCP-ALL cell lines.

### CD19-NKCEs promote NK cell–mediated lytic effects against primary BCP-ALL

**Figure 3.**
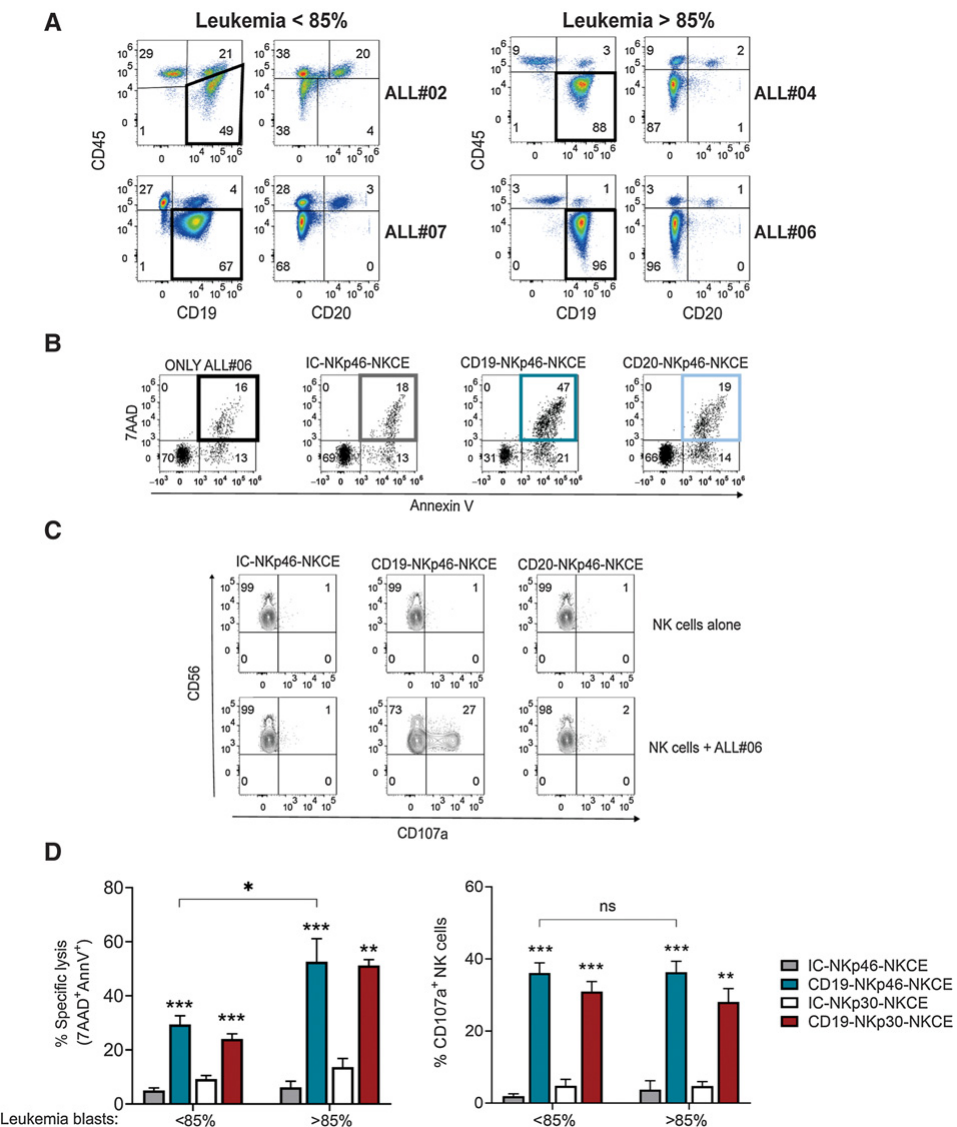
Effect of NKCEs targeting CD19 or CD20 on primary BCP-ALL cells. **A,** Phenotypic characterization via flow cytometry of four BCP-ALL primary leukemia samples, using CD45-APC-Vio770, CD19-PE-Cy7, and CD20-V450. **B,** 7AAD/AnnV staining of cells from sample ALL#06 cultured either alone (only ALL#06) or with resting NK cells from a representative healthy donor and the indicated NKCEs (10^0^ μg/mL). **C,** CD107a expression of NK cells cultured either alone or with cells from ALL#06 in the presence of IC-NKp46-NKCE, CD19-NKp46-NKCE, or CD20-NKp46-NKCE (10^0^ μg/mL). A representative experiment is shown. Numbers indicate the percentage of cells in each quadrant. **D,** Percent of specific lysis of CD19^+^ leukemia blasts (left) and CD107a degranulation (right) of resting NK cells from healthy donors (*n* = 3–6) upon coculture with primary leukemia blasts and NKCEs at 10^0^ μg/mL as indicated. Data obtained with target cells containing leukemia blasts <85% (ALL#02 and ALL#07) or >85% (ALL#04 and ALL#06) were pooled. Results from 2–5 independent experiments are reported. The incubation time for all the tests was 4 hours. Bar show mean ± SEM. Statistical significance: *, *P* ≤ 0.05; **, *P* ≤ 0.01; ***, *P* ≤ 0.001. Mann–Whitney test was used to calculate statistical differences.

Primary ALL blasts are known to be resistant to lysis by NK cells (19), particularly when NK cells are employed at resting state without any previous activation by cytokines (e.g., IL2 or IL15). Therefore, we investigated the effect of NKCEs against a panel of primary BCP-ALL cells obtained from pediatric patients at diagnosis. PBMCs or BMMCs were collected, and samples containing ≥50% leukemia blasts were selected. We analyzed the expression of CD45, CD19, CD20 (Fig. 3A), CD3 (Supplementary Fig. S4), and HLA class I molecules (Supplementary Fig. S5) by flow cytometry. BCP-ALL blasts were identified as CD45^dim^ cells that had high expression of CD19 and low (or almost negative) CD20 (Fig. 3A). Conversely, healthy B lymphocytes in patients were identified as CD45^bright^CD19^+^CD20^+^ cells. CD3^+^ cells were all CD45^bright^ (Supplementary Fig. S4). In sample ALL#06, 96% of BCP-ALL blasts were of the CD45^dim^CD19^+^CD20^−^ phenotype, and only 1% healthy B cells were present. In parallel, we also tested the efficacy of CD19-NKp46-NKCE and CD20-NKp46-NKCE at the optimal concentration (10^0^ μg/mL) to promote NK-cell activity against this target cell. The CD19-NKp46-NKCE was able to potentiate both NK-cell cytotoxicity (Fig. 3B) and CD107a degranulation (Fig. 3C) against BCP-ALL cells (ALL#06), but due to lack of CD20 expression, the CD20-NKp46-NKCE had no effect. Therefore, in further experiments with primary BCP-ALL cells, we utilized CD19-targeting NKCEs at the optimal concentration. Resting NK cells from healthy donors were challenged against different BCP-ALL primary blasts with CD19-NKCEs (or IC-NKCEs) in cytotoxicity and degranulation assays (Fig. 3D). Our data indicated that both CD19-NKp46-NKCE and CD19-NKp30-NKCE potentiated NK cell–mediated leukemia cell killing. A higher percentage of target cell lysis was obtained in samples with higher leukemia blast content. This difference was not observed in degranulation assays; the NKCEs induced efficient NK-cell activation against target cells comprised of either less than or more than 85% primary leukemia. These results provide evidence that CD19-NKCEs can engage resting NK cells and induce killing of primary BCP-ALL blasts.

### CD19-NKCEs enhance antileukemia activity of transplanted patient NK cells

**Figure 4.**
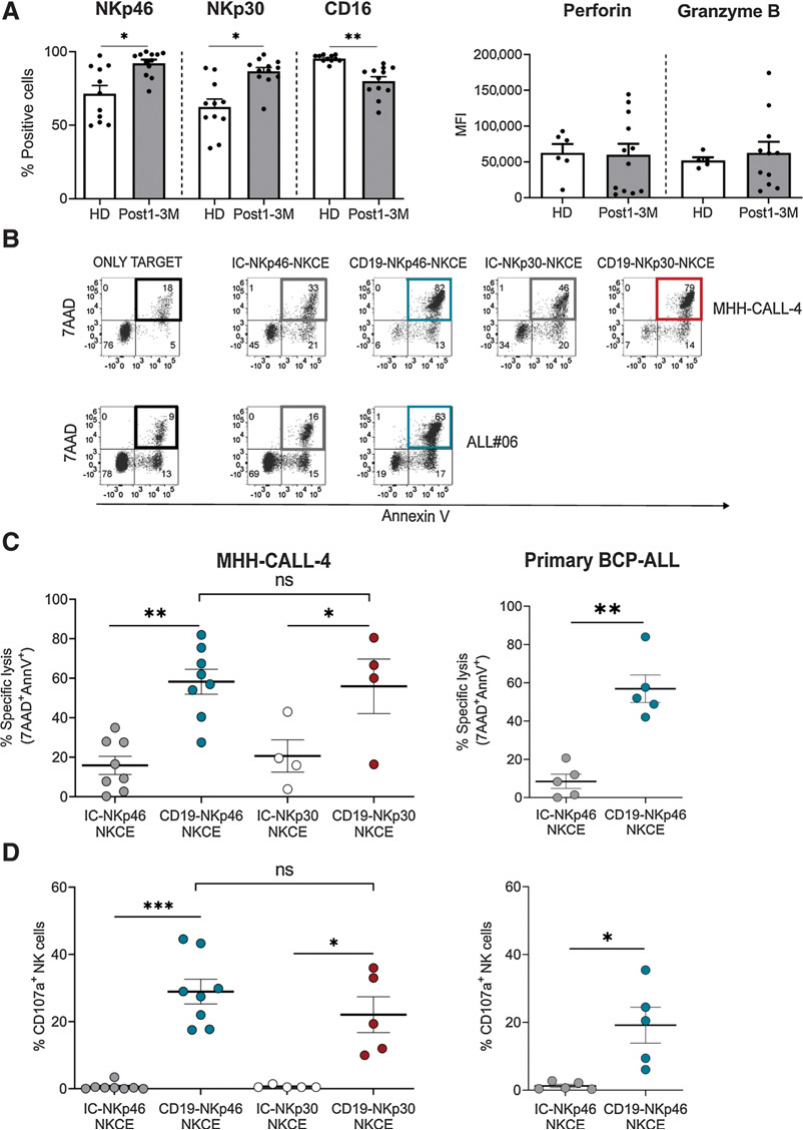
CD19-NKCEs efficiently promote NK-cell antileukemia activity in a transplantation setting. **A,** Evaluation of NKp46, NKp30, CD16, perforin, and granzyme B expression in NK cells from transplanted patients (Post1–3M; *n* = 12) and healthy donors (HD; *n* = 5–11). **B,** Representative experiment of cytototoxicity (7AAD/AnnV staining). MHH-CALL-4 or ALL#06 primary leukemia target cells were cultured either alone (only target) or with resting NK cells from a transplanted patient (3 months after haplo-HSCT) and the indicated NKCEs (10^0^ μg/mL). Numbers indicate the percentage of cells in each quadrant. Percent of specific lysis (7AAD/AnnV staining; **C**) and CD107a degranulation of resting NK cells (**D**) from transplanted patients against MHH-CALL-4 (left) or primary leukemia blasts (ALL#04 and ALL#06; right) in the presence of the indicated NKCE at 10^0^ μg/mL. Pooled data obtained with primary leukemia blasts are shown. Results from 5–8 independent experiments are reported. The incubation time for all the tests was 4 hours. Bar show mean ± SEM. Statistical significance: *, *P* ≤ 0.05; **, *P* ≤ 0.01; ***, *P* ≤ 0.001. Mann–Whitney test was used to calculate statistical differences.

We next evaluated whether the NKCEs could potentiate the anti-leukemia activity of NK cells derived from pediatric patients with leukemia after αβT/B-depleted haplo-HSCT. This transplantation setting is based on a graft manipulation strategy that allows the infusion of mature immune cells, mainly NK and γδT cells, in addition to hematopoietic stem cells. Indeed, engrafted mature and functional NK cells persist in the PB of recipients for at least one month, particularly when high numbers of NK cells have been infused within the graft (19, 36). At 1–3 months after transplantation, NK cells represented the most abundant lymphocyte subset; T (mainly γδT cells) cells were present, whereas B cells were almost absent (Supplementary Fig. S6; Supplementary Fig. S7). Using flow cytometry, we characterized NK cells in the patients' reconstituted repertoire (a representative case is shown in Supplementary Fig. S6B) and in healthy donors by evaluating surface expression of NKp46, NKp30, CD16, as well as intracellular perforin and granzyme B (Fig. 4A). Higher expression of NKp46 and NKp30 and lower expression of CD16 was observed in NK cells from transplanted patients compared with healthy donors. No significant differences were detected in perforin and granzyme B. Therefore, we exposed NK cells from these transplanted patients to the MHH-CALL-4 leukemia cell line and primary BCP-ALL cells containing >85% of blasts in the presence of NKCEs. CD19-NKp46-NKCE and CD19-NKp30-NKCE were equally able to enhance NK-cell killing (Fig. 4B and C) and degranulation (Fig. 4D), consistent with the data obtained with healthy donor–derived NK cells (Fig. 1C and Fig. 2B). We focused on the CD19-NKp46-NKCE and tested it in assays using primary BCP-ALL cells as target cells, due to limited cell availability from patient samples. The CD19-NKp46-NKCE enhanced NK-cell killing of BCP-ALL blasts (Fig. 4B–D), at levels comparable with those obtained with healthy NK cells (Fig. 3D). These data support that NK cells of αβT/B-depleted haplo-HSCT recipients are equipped with an adequate pattern of triggering receptors and lytic machinery, and that through NKCE engagement, these patient NK cells can efficiently kill BCP-ALL, both cell lines and primary blasts.

Considering that γδ T cells are highly represented in this transplantation setting and that these cells can express NKp30 upon stimulation with IL15 (42), we performed preliminary experiments using IL15–activated PBMCs from transplanted patients. Although all NK cells expressed NKp30, only few NKp30^+^ γδ T cells could be detected. Indeed, NK cells were efficiently triggered, whereas only little effect on γδ T degranulation was observed in the presence of the CD19-NKp30-NKCE and MHH-CALL-4 cells (Supplementary Fig. S7). This finding deserves further investigation.

### The effect of NKCEs can override HLA-specific inhibitory interactions

**Figure 5.**
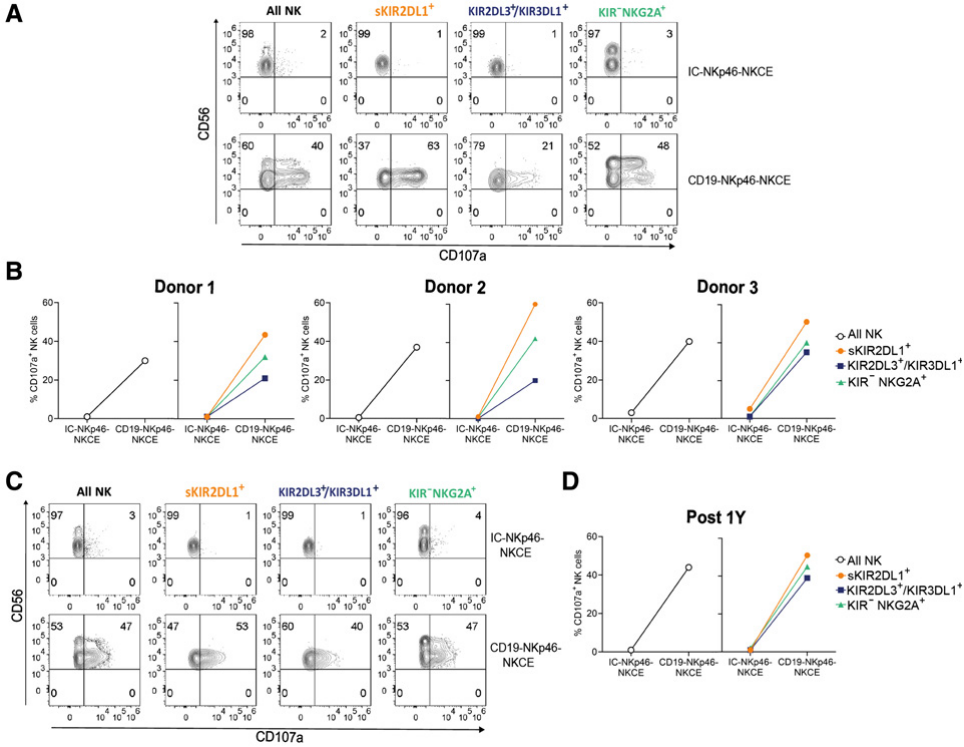
NKCEs override HLA-I inhibitory interactions. Degranulation activity of NK cells (All NK, gating on CD3^−^CD56^+^) and different NK-cell subsets [single (s)KIR2DL1^+^, KIR2DL3^+^/KIR3DL1^+^, and KIR^−^NKG2A^+^, identified by appropriate gating strategy reported in Supplementary Fig. S1] from healthy donors (*n* = 3) or transplanted patient (*n* = 1) upon 4-hour coculture with MHH-CALL-4 cells in presence of IC-NKp46- or CD19-NKp46-NKCE at 10^0^ μg/mL. Results from three independent experiments are reported. **A,** Contour plot of a representative donor showing the degranulation of different NK-cell subsets in the presence of the target cells and NKCEs. Numbers indicate the percentage of cells in each quadrant. **B,** Data from 3 healthy donors are reported. **C** and **D,** Degranulation of NK-cell subsets from 1 patient 1-year after haplo-HSCT. **C,** Contour plot of flow cytometry (raw data). **D,** Graphical representation.

We investigated whether the activating effect of NKCEs could be affected by HLA-specific inhibitory interactions between KIRs or NKG2A on effector NK cells and their cognate HLA class I ligands on BCP-ALL target cells. The MHH-CALL-4 cell line and primary leukemia blasts (gated as CD45^dim^ cells) expressed high HLA class I, which was, in most cases, similar to healthy counterparts (gated as CD45^bright^ cells; Supplementary Fig. S5). The analysis of KIR-Ls in MHH-CALL-4 cells indicated the presence of HLA-C alleles with only the C1 epitope, and the Bw4 epitope was carried by HLA-B (T^80^) and HLA-A alleles. We then obtained NK cells from selected samples from our cohort of haplo-HSCT donors and transplanted patients based on expression of C1, C2, and Bw4 epitopes and presence of a *KIR* A/A genotype. For the transplanted patients, we considered KIR-Ls and *KIR* genotype of the related donors. In these individuals, KIR2DL1^+^, KIR2DL3^+^, and KIR3DL1^+^ NK cells are educated, and the absence of KIR2DS1, KIR2DS2, and KIR3DS1 allows the lack of interference by these aKIRs in functional tests. NK cells from these donors and transplanted patients that have a C2 HLA-mismatch versus MHH-CALL-4 cells could contain the alloreactive subset (i.e., Allo-C2; ref. 36). NK cells from 3 healthy donors (Fig. 5A and B) and 1 patient who received αβT/B-depleted haplo-HSCT from an Allo-C2 donor (Fig. 5C and D) were selected. We evaluated NKCE-induced degranulation of different NK-cell subsets based on KIR and NKG2A expression: (i) expression of only KIR2DL3 and/or KIR3DL1 (i.e., KIR2DL3^+^/KIR3DL1^+^), which recognize C1 and Bw4 epitopes; (ii) expression of only CD94/NKG2A (i.e., KIR^−^NKG2A^+^), which recognizes HLA-E; or (iii) expression of only KIR2DL1 [i.e., single (s)KIR2DL1^+^, representing the Allo-C2 subset], which recognizes no HLA molecules on MHH-CALL-4 cells. These three NK-cell subsets were well represented in all individuals evaluated, including the donor-derived alloreactive subset in the reconstituted repertoire of the transplanted patient. We demonstrated that the CD19-NKp46-NKCE could promote the degranulation of all NK-cell subsets against MHH-CALL-4 cells (Fig. 5). These data indicated that the activation induced by the CD19-NKp46-NKCE against BCP-ALL cells can override the inhibitory signal(s) resulting from interaction(s) between iKIR/KIR-L and/or NKG2A/HLA-E. In all cases, the sKIR2DL1^+^ cells (with no inhibitory HLA-specific interactions) showed the highest activity, followed by KIR^−^NKG2A^+^ cells and KIR2DL3^+^/KIR3DL1^+^ cells, suggesting residual inhibition, with lower activity via NKG2A compared with the two KIRs recognizing HLA molecules on target cells.

## Discussion

NK cells are becoming more and more attractive in immunotherapy, particularly in the context of hematologic malignancies (21). Several strategies have been exploited to enhance their efficacy, often translating to NK cells the experience obtained through study of T cells. In addition to cell engineering using chimeric antigen receptor (CAR) constructs to produce CAR-NK cells (43, 44), different engagers have been developed to specifically arm NK cells and to selectively redirect them towards tumor cells. Here, we provided evidence that NKCEs, especially the CD19-NKp46-NKCE, could engage resting NK cells derived from healthy controls and transplanted patients. These molecules promoted the lysis of BCP-ALL cells, which otherwise would be resistant. Indeed, primary pediatric BCP-ALL blasts are characterized by the expression of CD19, whereas CD20 is virtually negative, leading to lower interest in CD20-NKCEs, as well as possible therapies with anti-CD20 like rituximab. Considering the known surface NK-cell receptor ligands, BCP-ALL cells generally have high HLA class I, presence of the DNAM-1 ligand Nectin-2, and absence of all NKG2D ligands (36, 41). Functional data suggest that BCP-ALL cells also express NKp46 ligands, although their molecular identification has still not been achieved (36, 41).

Here, we described that resting NK cells could kill NALM-16 BCP-ALL cells, which carry a hemizygous HLA haplotype and express many surface activating receptor ligands (i.e., PVR, Nectin-2, MICA, and ULBP1–3; ref. 36). In contrast, MHH-CALL-4 cells resembled primary BCP-ALL blasts, both in terms of phenotypic features and resistance to lysis by resting NK cells. Indeed, we previously showed that primary leukemia blasts could be killed only by cytokine-activated NK cells, particularly in case of alloreactive NK cells that are not inhibited by HLA class I on target cells (19, 36). In T cell–depleted haplo-HSCT, KIR/KIR-L mismatch in GvH direction associates with a reduced risk of disease recurrence in patients with acute leukemia, due to an alloreactive NK cell–mediated GvL effect, and is observed in both adult and pediatric patients (18, 45). Thus, we consider NK alloreactivity of first priority in donor selection criteria in the context of αβT/B cell–depleted haplo-HSCT to cure pediatric patients with high-risk leukemia, including BCP-ALL. This transplantation platform allows the engraftment, together with HSC, of mature NK and γδ T cells that persist in the patient circulation as immunocompetent cells (36, 46). αβT/B cell–depleted haplo-HSCT represents an important therapeutic option for patients with BCP-ALL, which is the most common pediatric leukemia.

We tested the *in vitro* effect of NKCEs on NK cells derived from donors and posttransplant patients. The ability of the NKCEs to ligate both CD16A and NKp46 is relevant in this clinical context because NK cells in the reconstituted repertoire (contains NK cells at different stages of maturation) can present higher proportions of CD56^bright^CD16^dim/neg^ cells compared with healthy PB NK cells. However, we found that both CD56^bright^ and CD56^dim^ NK cells usually expressed high NKp46. It is also worth mentioning that an additional donor selection criterion accounts on NCR^bright^ phenotype of NK cells, possibly related to high antileukemia activity (36). We documented that the CD19-NKp46-NKCE was efficient in triggering the lysis of primary BCP-ALL blasts, even by resting NK cells derived from transplanted patients. These experiments are challenging because few PBMCs are obtained from small volumes of children blood samples. In degranulation assays, we circumvented the problem of NK-cell purification and used total PBMCs from patients at early timepoints after transplant, which were characterized by a predominance of NK cells and absence of B cells. We also provided evidence that the effect of NKCEs could override the inhibition delivered by iKIRs and/or NKG2A upon interaction with their ligands, although the alloreactive subset always exerted the highest activity.

NKp30-NKCEs were as efficient as NKp46-NKCEs, consistent with their equal expression and function on NK cells (6). Bispecific immuno-ligands, via NKp30 engagement, are more efficient against EGFR-overexpressing tumor cells than clinically approved cetuximab (31). NKp30 expression has been also described on γδ T and CD8^+^ T cells upon culture in IL15 (42, 47). Considering that γδ T cells are well represented in αβT/B cell–depleted haplo-HSCT, these lymphocytes might also benefit from NKp30-NKCEs.

The 161519 TriKE can induce NK-cell proliferation, is more effective than rituximab in inducing the *in vitro* killing of the Raji lymphoma cell line and primary chronic lymphocytic leukemia (CLL) targets by healthy NK cells, and can restore functionality of NK cells from patients with CLL (27). In xenograft models involving human PBMCs and Namalwa lymphoma cells, treatment with the 161519 TriKE induces sustained antitumor activity compared with the 1619 BiKE, whose activity could be enhanced by combined use of IL2 (48). NKCEs targeting CD19 or CD20 are demonstrated to be more potent *in vitro* than rituximab against Daudi lymphoma cell line (34), as was demonstrated in our present study using BCP-ALL cell lines. In mouse models, NKCEs targeting CD20 can control the growth of Raji lymphoma cell line, show *in vivo* pharmacokinetics similar to obinutuzumab, and have no off-target effects (34). Our *in vitro* data are encouraging to envisage the use of the CD19-NKp46-NKCE in clinical practice to fight BCP-ALL in children with relapsed/refractory disease, including relapse after allogeneic HSCT. In the case of relapse after haplo-HSCT, the antileukemia activity of NK cells already present in the patient reconstituted repertoire can be further supported by infusing NK cells derived from the same haploidentical donor of the graft, possibly also benefiting from NK alloreactivity. NKCEs can be used together with adoptive NK-cell transfer. A more effective approach might require the infusion of cytokine-activated NK cells that are *ex vivo* precomplexed with the engager, as has been proposed for AFM13 and IL12/15/18 preactivated NK cells to treat CD30^+^ malignancies (49). Overall, this approach using the CD19-NKp46-NKCE and NK cells is safe and feasible and might complement other existing treatments for BCP-ALL, such as blinatumomab (acts on T cells; refs. 50–54).

## Supplementary Material

Supplementary Data
