# Supplementary material for: Exploiting Natural Killer Cell Engagers to Control Pediatric B-cell Precursor Acute Lymphoblastic Leukemia
Source: Cancer Immunol Res. 2022 Mar 3;10(3):291–302. doi: 10.1158/2326-6066.CIR-21-0843 (PMC9662914; doi:10.1158/2326-6066.CIR-21-0843)
Supplement: Supplementary Data [file cir-21-0843_supplemental_cir-21-0843_suppst1-sf-7.pdf]

Supplementary Table S1. Antibodies used in cytofluorimetric analysis

Specificity	Fluorochrome	Clone	Isotype	Amount/ sample	Catalog no.	Vendor
CD3	PE-CF594	UCHT1	IgG1	3 µL	562280	BD Bioscience, San Jose, CA, USA
CD3	BV510	UCHT1	IgG1	3 µL	563109	BD Bioscience, San Jose, CA, USA
CD19	PE-CF594	HIB19	IgG1	3 µL	562294	BD Bioscience, San Jose, CA, USA
CD16	FITC	3G8	IgG1	3 µL	555406	BD Bioscience, San Jose, CA, USA
CD56	BV421	NCAM 16.2	IgG2b	1 µL	562751	BD Bioscience, San Jose, CA, USA
KIR2DL2/S2/L3	FITC	CHL	IgG2b	4 µL	559784	BD Bioscience, San Jose, CA, USA
CD107a	FITC	H4A3	IgG1	5 µL	555800	BD Bioscience, San Jose, CA, USA
CD107a	PE	H4A3	IgG1	5 µL	555801	BD Bioscience, San Jose, CA, USA
IFN-γ	PE	B27	IgG1	0,5 µL	554701	BD Bioscience, San Jose, CA, USA
CD20	V450	L27	IgG1	3 µL	561164	BD Bioscience, San Jose, CA, USA
CD20	Unconjugated	L27	IgG1	2,5 µg/mL	347670	BD Bioscience, San Jose, CA, USA
CD56	PE-Cy7	N901	IgG1	3 µL	A21692	Beckman Coulter, Brea, CA, USA
KIR2DL1/S1, KIR2DL3*005	PE	EB6B	IgG1	5 µL	A09778	Beckman Coulter, Brea, CA, USA
KIR2DL1/S1, KIR2DL3*005	PE-Cy7	EB6B	IgG1	3 µL	A66899	Beckman Coulter, Brea, CA, USA
KIR2DL2/S2/L3	PE-Cy7	GL183	IgG1	3 µL	A66901	Beckman Coulter, Brea, CA, USA
NKG2A	APC	Z199	IgG2b	3 µL	A60797	Beckman Coulter, Brea, CA, USA
TCR PAN γδ	FITC	IMMU510	IgG1	5 µL	B49175	Beckman Coulter, Brea, CA, USA
CD3	VioBlue	BW264/56	IgG2a	0,6 µL	130-113-133	Miltenyi Biotech, Bergisch Gladbach, Germany
CD45	APC-Vio 770	5B1	IgG2a	0,6 µL	130-113-115	Miltenyi Biotech, Bergisch Gladbach, Germany
NKp46 (CD335)	PE	9E2	IgG1	3 µL	130-092-607	Miltenyi Biotech, Bergisch Gladbach, Germany
KIR3DL1	FITC	DX9	IgG1	0,4 µL	130-123-668	Miltenyi Biotech, Bergisch Gladbach, Germany
KIR3DL1	PE-Vio 770	DX9	IgG1	2 µL	130-099-959	Miltenyi Biotech, Bergisch Gladbach, Germany
KIR2DL1, 2DS5	PE	143211	IgG1	5 µL	FAB1844P	R&D systems, Minneapolis, MN, USA

Granzyme B	Alexa Fluor 647	GB11	IgG1	2 µL	515406	Biolegend, San Diego, CA, USA
Isotype control	Alexa Fluor 647	MOPC-21	IgG1	2 µL	400136	Biolegend, San Diego, CA, USA
NKp30 (CD337)	Alexa Fluor 647	P30-15	IgG1	3 µL	325212	Biolegend, San Diego, CA, USA
Perforin	PE	δG9	IgG2b	1 µL	358-050	Ancell, Stillwater, MN, USA
Isotype control	PE	BPC 4	IgG2b	1 µL	284-050	Ancell, Stillwater, MN, USA
CD19	PE-Cy7	SJ25C1	IgG1	3 µL	25-0198-42	Thermo Fisher, Waltham MA, USA
HLA class I	Unconjugated	W6/32	IgG2a	2 µg/mL		Our Laboratory
CD19	Unconjugated	BU19	IgG1	2 µg/mL		Our Laboratory

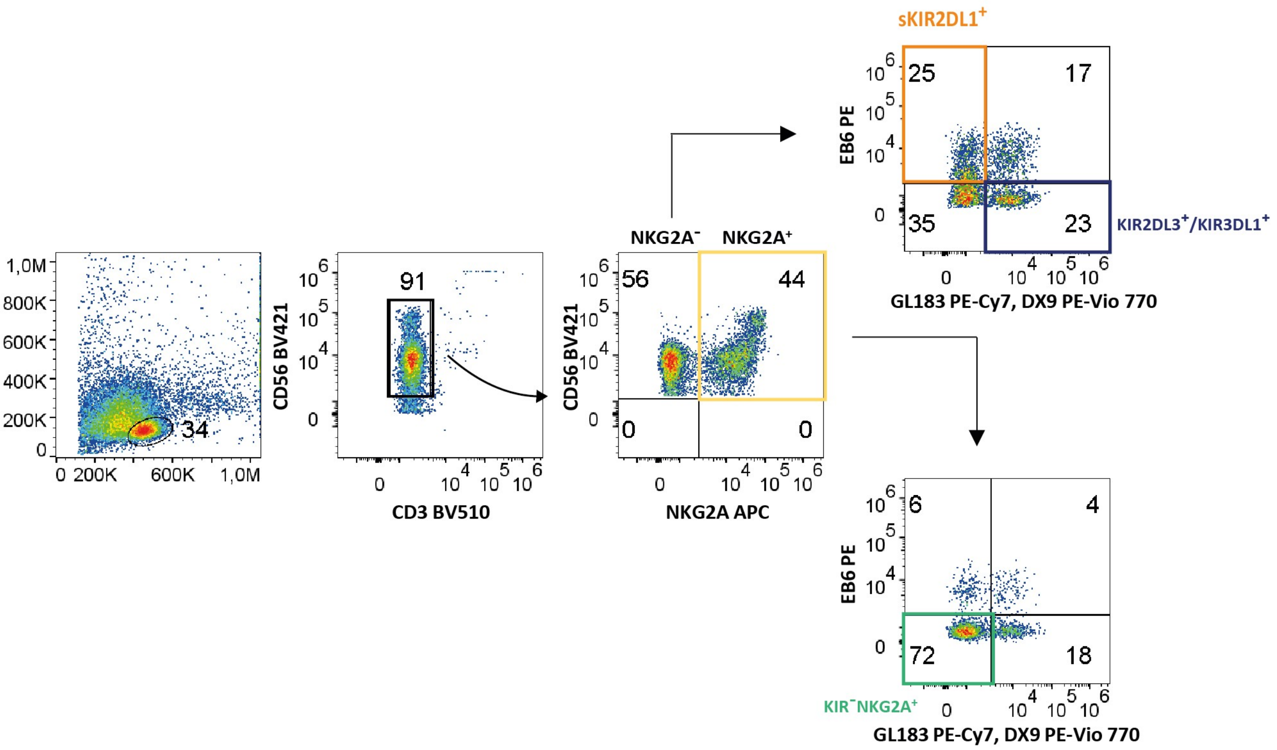

Supplementary Figure S1. Gating strategy to define NK cell subsets in degranulation assay. Purified NK cells from donors characterized by an A/A *KIR* genotype were stained with the following mAb combination to identify sKIR2DL1^{+} (orange), KIR2DL3^{+}/KIR3DL1^{+} (blue), and KIR^{-}NKG2A^{+} (green) NK cell subsets: CD3-BV510, CD56-BV421, NKG2A-APC, EB6- or 143211-PE, GL183-PE-Cy7, DX9-PE-Vio 770 and CD107a-FITC. In some experiments, the mAb combination was modified using CD107a-PE, and, consequently, EB6-PE-Cy7, CHL- and DX9-FITC were used.

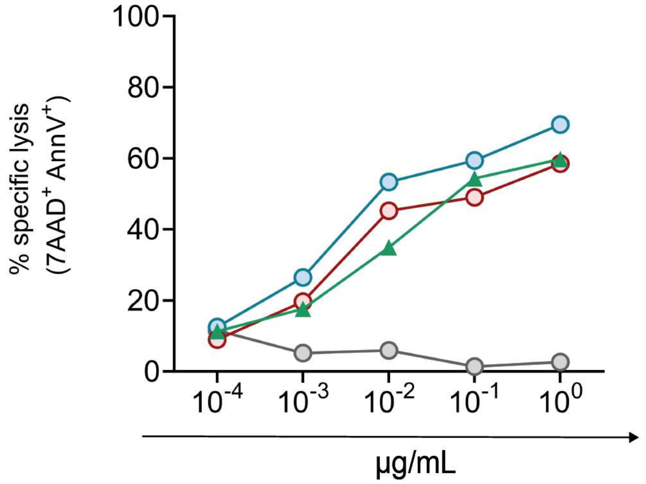

Supplementary Figure S2. Cytotoxicity assay based on 7AAD/AnnV staining presents similar results to ^{51}Cr-release assay (reported in Fig. 1B). Resting NK cells from a representative healthy donor were tested against MHH-CALL-4 using different concentrations of CD20-NKCEs or rituximab. E:T ratio 10:1.

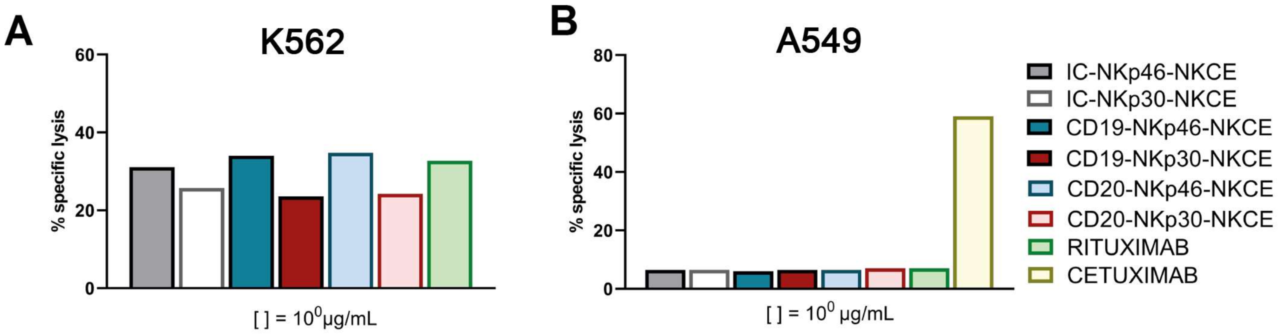

Supplementary Figure S3. CD19- and CD20-NKCEs do not enhance the killing of CD19^{-} and CD20^{-} cell lines: K562 **(A)** and A549 **(B)**. E:T ratio 10:1 (K562) and 5:1 (A549). Results from a representative donor are shown.

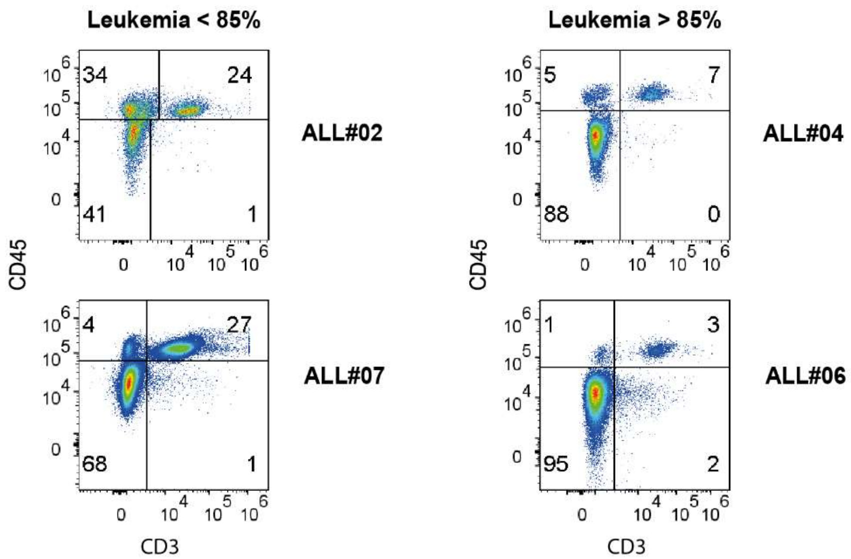

Supplementary Figure S4. Analysis of CD45 and CD3 expression on samples obtained from four leukemia patients at disease onset. $CD3^{+}$ cells, which are all $CD45^{bright}$, represent the healthy counterpart. Numbers indicate the percentage of cells in each quadrant.

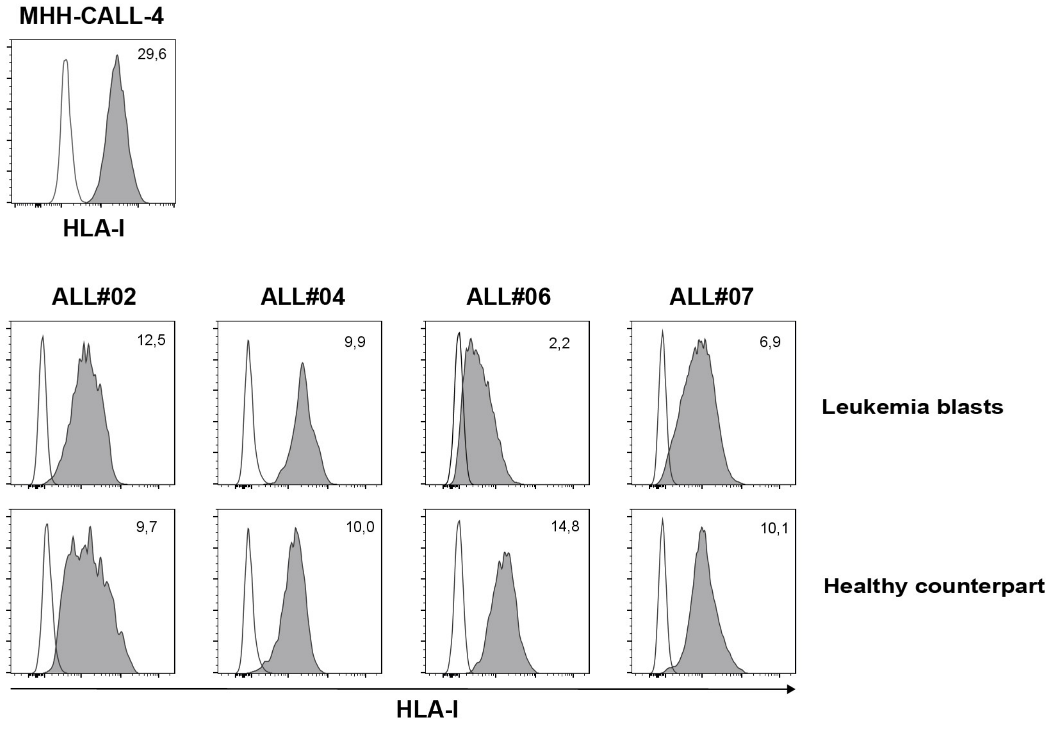

Supplementary Figure S5. Analysis of HLA class I (HLA-I) expression on MHH-CALL-4 cell line and samples obtained from four leukemia patients at disease onset. Immunofluorescence was performed using W6/32 mAb in combination with anti-IgG FITC secondary reagent (gray histograms). Negative controls with the secondary reagent were also performed (white histograms). The expression of HLA-I on leukemia blasts ($CD45^{dim}$) is compared to the healthy counterpart ($CD45^{bright}$) by an appropriate gating strategy. Numbers indicate the staining index (SI).

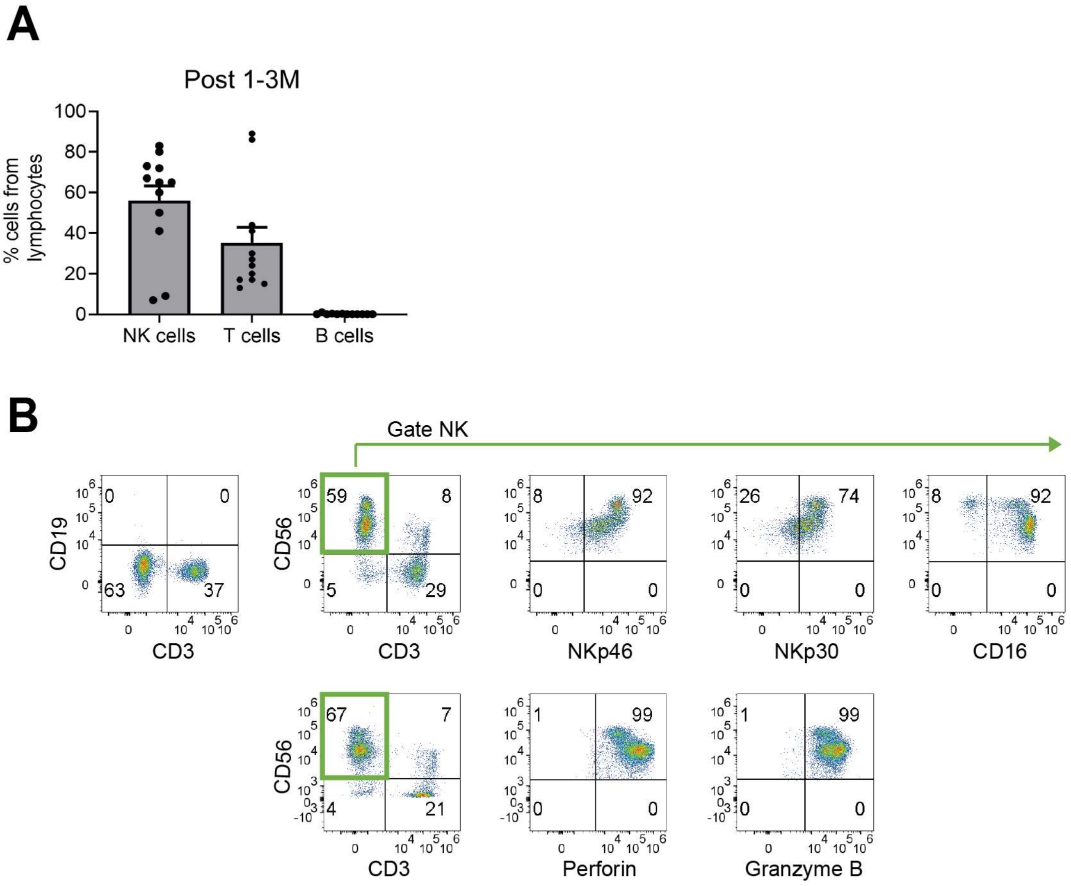

Supplementary Figure S6. Immunological reconstitution in patients at early time points after αβT/B-depleted haplo-HSCT. A) % of NK cells (CD3^{-}CD56^{+}) T cells (CD3^{+}CD56^{-}), and B cells (CD19^{+}CD3^{-}) in patients at 1 or 3 months after haplo-HSCT (Post 1-3M). **B)** Representative flow cytometry analysis of PBMC of a patient (post-3M): identification of different lymphocyte subsets, and characterization of either surface expression of NKp46, NKp30, and CD16, or intracellular staining of perforin and granzyme B on NK cells (gating on CD3^{-}CD56^{+}). The following mAb combinations were used: i) CD19-PE-CF594, CD3-VioBlue, CD56-PE-Cy7, NKp46-PE, NKp30-Alexa Fluor 647, and CD16-FITC;ii) CD3-PE-CF594, CD56-BV421, perforin-PE, and granzyme B-Alexa Fluor 647. Numbers indicate the percentage of cells in each quadrant.

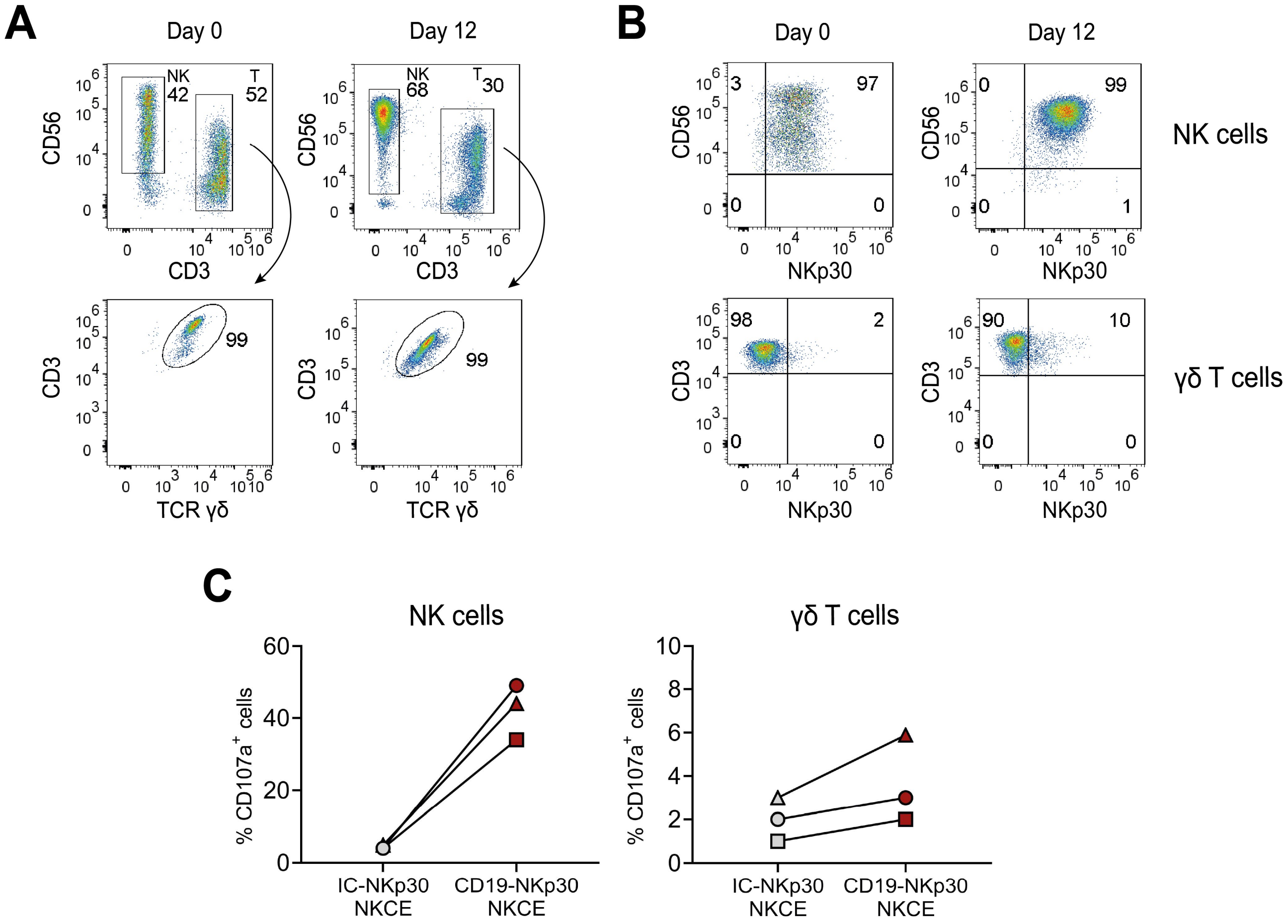

Supplementary Figure S7. Effect of CD19-NKp30-NKCE on γδ T cells. A-B) Flow cytometry analyses performed on PBMC either freshly isolated (day 0) or cultured 12 days with IL-15 at 10 ng/mL (day 12) in a representative patient at 1 month after haplo-HSCT. **A)** % of NK cells ($CD3^{-}CD56^{+}$) and T cells ($CD3^{+}$) of lymphocytes at day 0 and day 12. γδ T cells were identified gating on $CD3^{+}$ cells and represented the totality of the $CD3^{+}$ population (lower panels). The mAb combination was: CD3-PE-CF594, CD56-PE-Cy7, and TCR PAN γδ-FITC. **B)** Analysis of NKp30 expression on NK cells ($CD3^{-}CD56^{+}$) and γδ T cells ($CD3^{+}$) at day 0 and day 12. The mAb combination was: CD3-PE-CF594, CD56-PE-Cy7, TCR PAN γδ-FITC, and NKp30-Alexa Fluor 647. In the staining of samples at day 12, we also used Live/Dead Fixable Aqua stain to exclude dead cells. Numbers indicate the percentage of cells in each gate. **C)** CD107a degranulation assay of IL-15 cultured (day 12) NK cells or γδ T cells from three transplanted patients against MHH-CALL-4 in the presence of the indicated NKCEs at 10^{0} µg/mL. NK cells were identified gating on $CD3^{-}CD56^{+}$ cells, whereas γδ T cells gating on $CD3^{+}$ TCR $γδ^{+}$ cells. The triangle corresponds to the representative patient shown in panels A and B.
